# *Stroke mimic* tras picadura de avispa en niño con síndrome de Charcot-Marie-Tooth ligado al cromosoma X tipo 1

**DOI:** 10.23938/ASSN.1157

**Published:** 2026-07-21

**Authors:** Alicia Hernando Larroy, Alicia Frías Herrero, José Luis Peña Segura, Daniel Palanca Arias

**Affiliations:** 1 Servicio de Pediatría Hospital Universitario Miguel Servet Zaragoza España; 2 Unidad de Neuropediatría Hospital Universitario Miguel Servet Zaragoza España; 3 Unidad de Cuidados Intensivos Pediátricos y Unidad de Cardiología Pediátrica Hospital Universitario Miguel Servet Zaragoza España

**Keywords:** Proteína GJB1, Enfermedad de Charcot- Marie-Tooth, Polineuropatía, Picadura de Insecto, Manifestaciones Neurológicas, Gap Junction beta-1 Protein, Charcot-Marie- Tooth Disease, Polyneuropathy, Insect Bites and Stings, Neurologic Manifestations

## Abstract

El síndrome Charcot-Marie-Tooth tipo X1 (CMTX1) es una neuropatía motora y sensitiva hereditaria, causada por mutaciones en el gen GJB1 del cromosoma X. Aunque infrecuentes, la literatura describe cuadros tipo *stroke mimic* en pacientes CMTX1 en relación a episodios de estrés, con hallazgos característicos en la resonancia magnética y resolución espontánea completa.

Presentamos el caso de un adolescente varón de 13 años con CMTX1, sin clínica neurológica previa, que tras picadura de avispa presentó cuadro de hemiparesia derecha, disartria y desviación bucal. Tras activación del código ictus, la resonancia magnética cerebral mostró lesiones hiperintensas bilaterales en centros semiovales, sin signos de trombosis. Sería el primer caso en un paciente pediátrico con CMTX1 con picadura de insecto como desencadenante del cuadro que mimetizó un ictus. Consideramos la importancia de, ante la duda, activar el código ictus pediátrico y realizar resonancia magnética para confirmar el diagnóstico y descartar otros procesos.

## Abstract (en)

Charcot-Marie-Tooth syndrome type X1 (CMTX1) is an inherited motor and sensory neuropathy caused by mutations in the GJB1 gene located in the X chromosome. Although uncommon, stroke-like episodes have been reported in patients with CMTX1, typically triggered by stress and characterized by distinctive magnetic resonance imaging findings with complete spontaneous resolution.

We report the case of a 13-year-old boy with CMTX1 and no prior neurological symptoms who developed right hemiparesis, dysarthria, and facial deviation following a wasp sting. After activation of the stroke code, brain magnetic resonance revealed bilateral hyperintense lesions in the centrum semiovale without evidence of thrombosis. To our knowledge, this is the first paediatric case of CMTX1 in which an insect sting acted as a trigger, mimicking stroke. Prompt activation of stroke protocols and neuroimaging is essential to establish the diagnosis and exclude other conditions.

## INTRODUCCIÓN

El síndrome de Charcot-Marie-Tooth (CMT) es una polineuropatía hereditaria motora y sensitiva que cursa con debilidad muscular distal, afectación sensitiva, hiporreflexia y pies cavos. La clasificación clínica y genética del CMT es muy amplia. El CMT ligado al cromosoma X tipo 1 (CMTX1) (#302800) es el segundo tipo más frecuente (10-20% de los casos) y se produce por una mutación en el gen GJB1, que codifica la proteína beta 1 de uniones gap, llamada conexina 32^1^.

Aunque el CMTX1 es un trastorno del sistema nervioso periférico, se han descrito casos de pacientes que presentan episodios de focalidad neurológica con resolución espontánea, simulando un ictus isquémico^2^^-^^5^. Estos episodios motivan la activación del código ictus y la realización de pruebas de imagen urgentes, suponiendo un reto diagnóstico. Conocer esta entidad permite evitar estudios invasivos adicionales y tratamientos iatrogénicos, como en el caso del adolescente presentado.

## CASO CLÍNICO

**Figura 1:**
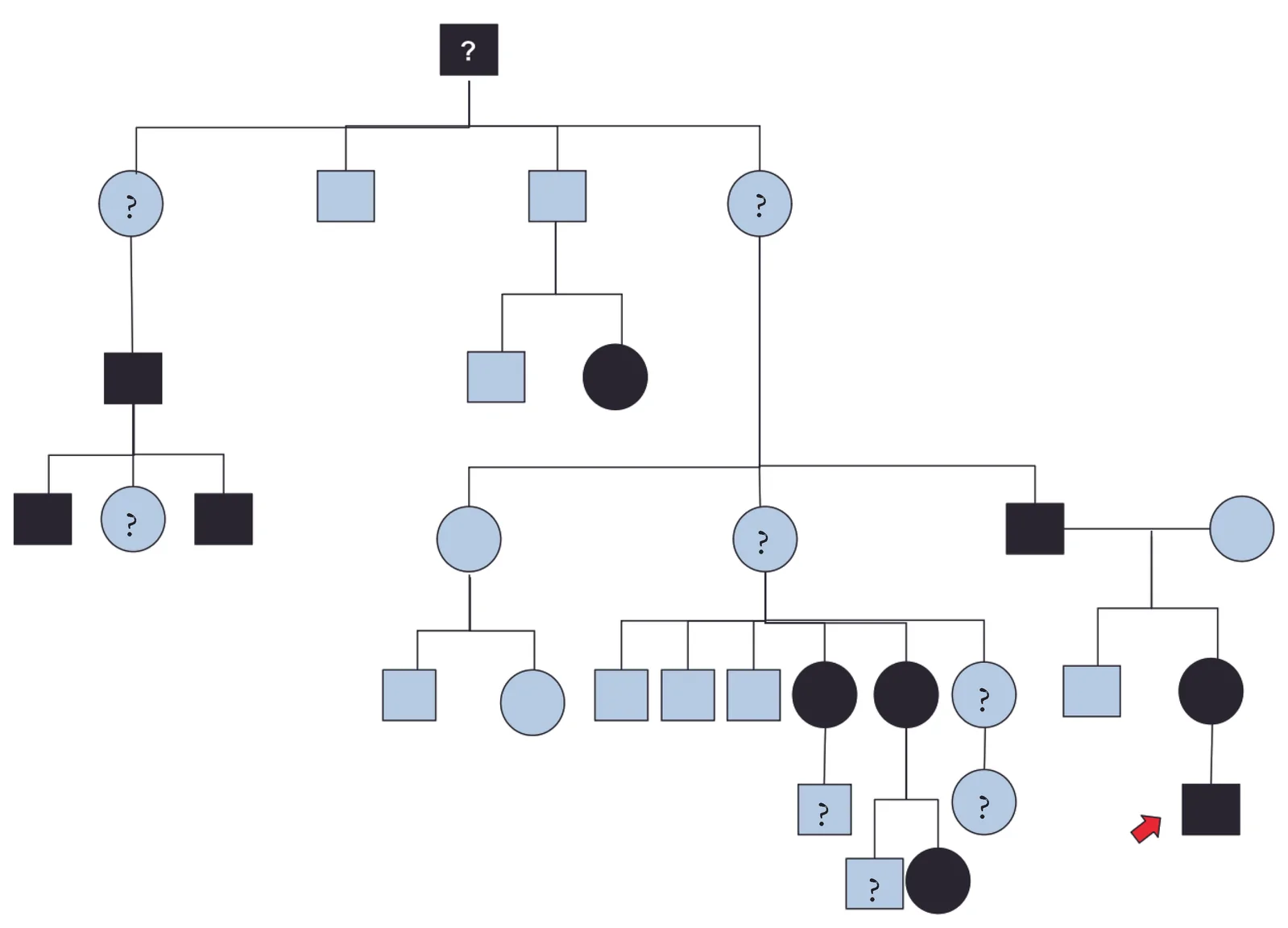
Árbol genealógico del paciente (flecha). En negro, los familiares afectados de CMTX1. Cuadrado: hombre; círculo: mujer.

Se presenta el caso de un adolescente varón de 13 años que consultó en su centro de salud por presentar aparición brusca de parestesias en mejilla derecha tras haber sufrido una picadura de avispa en el pie derecho ocho horas antes. En antecedentes personales de interés destacaba el diagnóstico genético de CMTX1: el paciente era portador en hemicigosis de la mutación c.20A>G del exón 1 del gen GJB1, presente en varios miembros de la rama materna de su familia (Figura 1).

**Figura 2:**
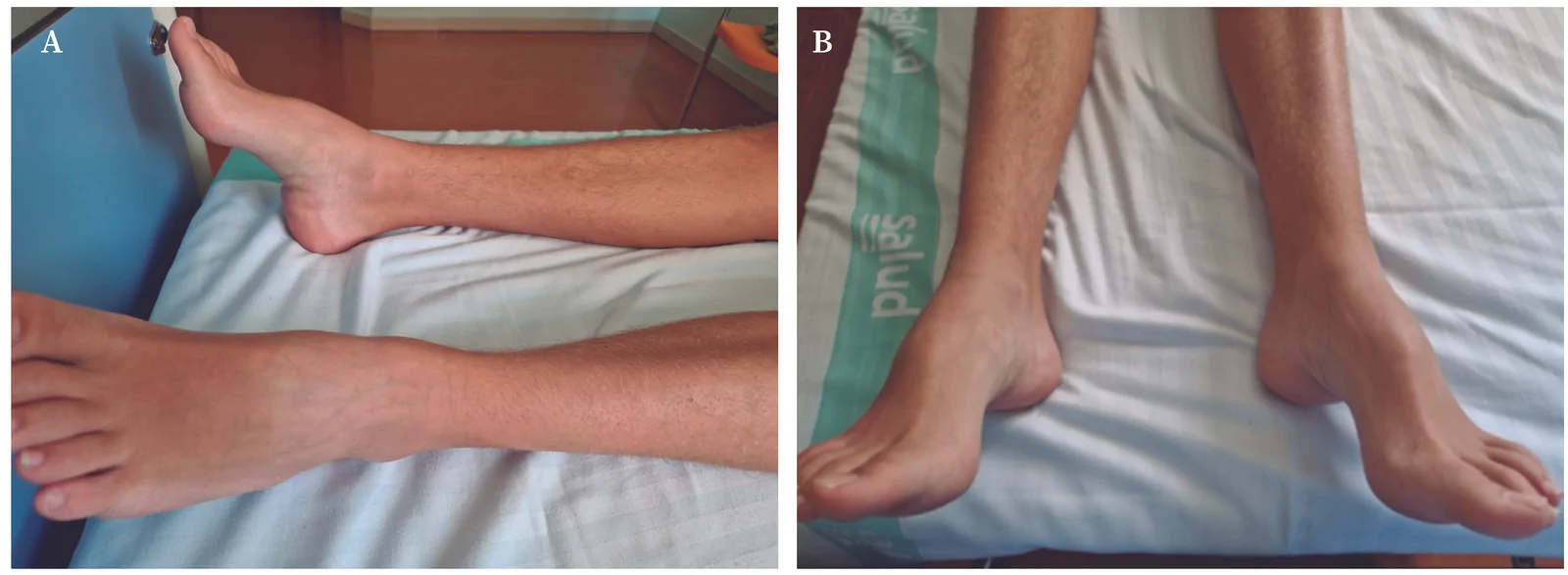
Vista lateral (**A**) y frontal (**B**) de los pies cavos del paciente.

En la exploración neurológica basal destacaron pies cavos (Figura 2), debilidad muscular en flexores de ambos pies, y reflejos rotulianos y aquíleos débiles. Se administró una dosis de corticoide oral y, ante la presencia de focalidad neurológica, se trasladó a hospital de área.

Durante la exploración física a su llegada al hospital de área se observó hemiparesia derecha con desviación de comisura bucal a la izquierda, borramiento de surco nasogeniano derecho y disartria, por lo que se activó el código ictus y se trasladó a la Unidad de Cuidados Intensivos Pediátricos (UCIP).

**Figura 3:**
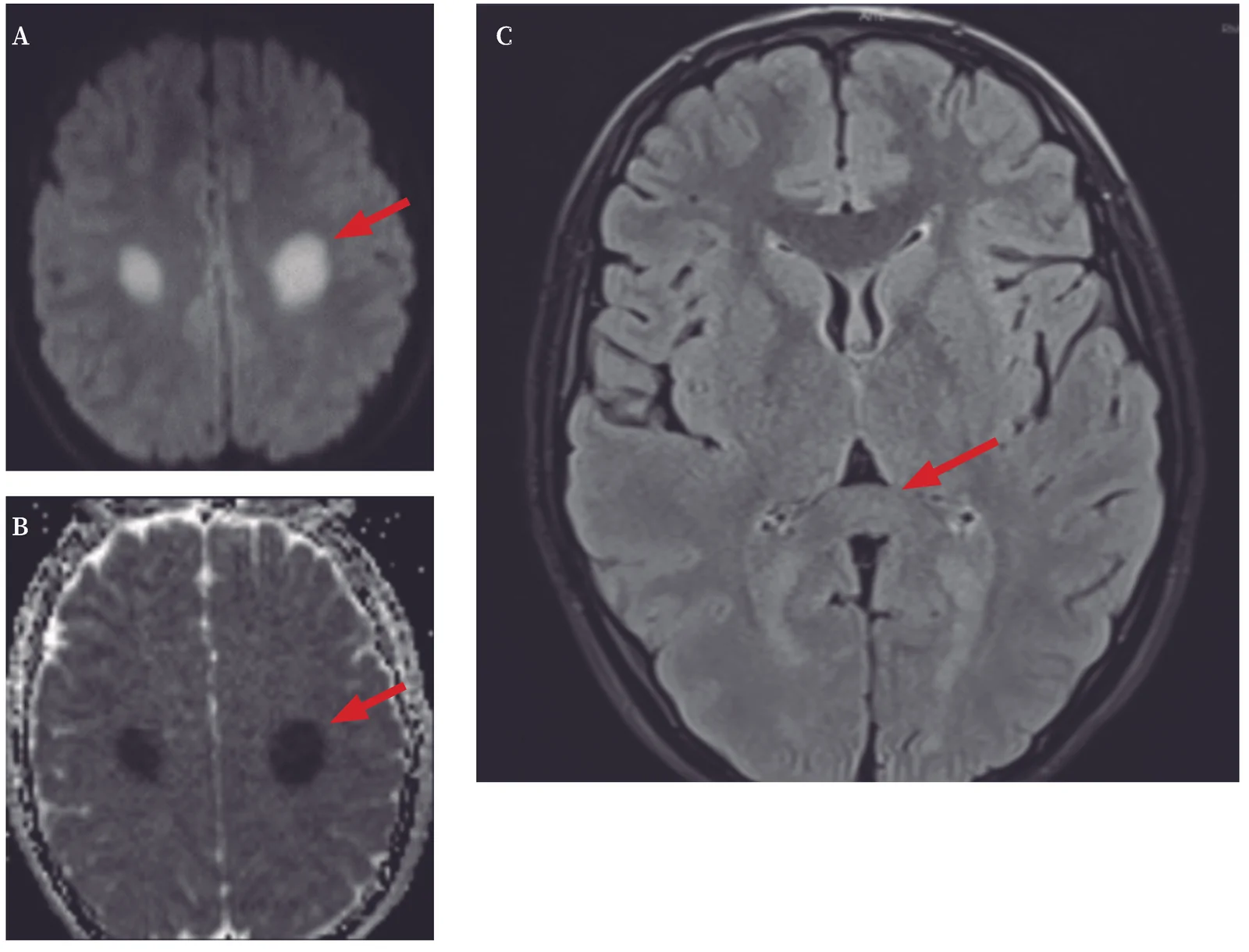
Resonancia magnética cerebral. Ambos centros semiovales (flecha) mostraron hiperintensidad en la secuencia DWI (imagen ponderada por difusión) (**A**), e hipointensidad en el mapa de coeficiente de difusión aparente (ADC) (**B**). **C**. La secuencia FLAIR T2 muestra alteración de señal que afecta al esplenio del cuerpo calloso (flecha).

Durante su evaluación en la UCIP se le realizó una resonancia magnética cerebral urgente, que mostró lesiones hiperintensas en T2 a nivel de ambos centros semiovales y de esplenio del cuerpo calloso (Figura 3), y una angiotomografía computarizada (angioTC) de troncos supraaórticos, sin alteraciones. Cuatro horas tras el inicio de la clínica neurológica se observó una clara mejoría. Aunque en la exploración persistía un sutil borramiento del surco nasogeniano derecho y disartria leve, el resto de la exploración no mostró alteraciones relevantes.

El paciente mostró mejoría clínica progresiva durante un tiempo de evolución superior a cuatro horas, por lo que -junto a las imágenes radiológicas que descartaban patología trombótica- se desestimó iniciar tratamiento de reperfusión. Ante la posible causa inflamatoria, se optó por administrar megabolus de corticoides a 30 mg/Kg/día y tratamiento antiagregante con ácido acetilsalicílico (100 mg/24 horas).

Tras revisar la literatura existente, donde se describen episodios tipo *stroke mimic* en pacientes con el síndrome de CMTX1 con resolución espontánea de los síntomas, se decidió suspender el tratamiento corticoide y antiagregante. A las 24 horas del ingreso, el paciente recuperó su situación neurológica basal y fue dado de alta con seguimiento mediante resonancia magnética y controles clínicos en su centro de atención primaria.

## DISCUSIÓN

El síndrome de Charcot-Marie-Tooth ligado al cromosoma X (CMTX1) está causado por una mutación en el gen GJB1, localizado en el brazo corto de dicho cromosoma, que codifica la proteína β-1 de las uniones *gap* o conexina 32^1^^-^^6^. La conexima 32 está implicada en la formación de hemicanales de conexina entre las capas de la vaina de mielina del axón, permitiendo la difusión de iones entre el citoplasma alrededor del núcleo de la célula de Schwann y el citoplasma de las capas más internas de la mielina que rodean el axón^1^. La disfunción de la conexina 32 afecta a la difusión de iones que permiten la propagación del potencial de acción. Esta proteína se expresa tanto en las células de Schwann como en los oligodendrocitos, lo que podría explicar que pacientes con una enfermedad del sistema nervioso periférico puedan sufrir episodios de afectación del sistema nervioso central^1^^,^^4^^,^^7^.

Se han descrito casos de pacientes con CMTX1 que sufren episodios de inicio agudo de focalidad neurológica transitoria tipo *stroke mimic*, con resolución espontánea y recuperación completa^2^^,^^4^^,^^5^. Estos eventos se han observado casi exclusivamente en varones de entre 5 y 26 años, mientras que es excepcional en mujeres, ya que portan la mutación en heterocigosis^2^. Entre los síntomas más frecuentes aparecen debilidad facial, lingual o de extremidades en un 93% de los casos, seguida de disartria o disfagia en un 83%. La duración de los síntomas es muy variable, desde tres minutos hasta seis meses. Un 80% de los casos presenta más de un episodio^2^^,^^3^^,^^8^^,^^9^.

Los casos publicados describen desencadenantes como: procesos infecciosos, altitud, falta de sueño, ejercicio intenso o hiperventilación^2^^,^^10^^,^^11^. En nuestro paciente, la situación de estrés metabólico relacionada con la picadura de avispa podría haber desencadenado el episodio, aunque no está descrita en la literatura.

El paciente presentó clínica neurológica unilateral, mientras que la afectación era bilateral en la resonancia magnética cerebral. Este es un hecho común a todos los casos descritos^3^^,^^9^. En todos ellos, la resonancia magnética mostraba imágenes similares, con afectación bilateral de centro semioval y esplenio del cuerpo calloso con señal hiperintensa en T2 y FLAIR.

Ante un episodio de inicio agudo de focalidad neurológica en un paciente con CMTX1, estaría indicado activar el código ictus y realizar RM cerebral. En los diferentes casos revisados no se encontró diferencia significativa entre los pacientes tratados o no con corticoesteroides, siendo lo más frecuente la resolución espontánea de los síntomas^2^. Además, habrá que considerar el diagnóstico diferencial con accidentes isquémicos transitorios, cuadros metabólicos o procesos inflamatorios como la encefalitis aguda diseminada (EMAD)^10^.

Aunque es probable que las lesiones centrales de CMTX1 sean reversibles, está descrito que pueden persistir durante varios años después de un evento, por lo que se aconsejan controles de imagen posteriores^11^.

En pacientes pediatricos con CMTX1 con síntomas neurológicos compatibles con episodio tipo *stroke mimic* y persistencia de clínica neurológica en el momento de la valoración, debemos activar el código ictus con diligencia, a pesar de que -con frecuencia- podemos enfrentarnos a un *stroke mimic*. Tener en cuenta esta entidad evita estudios invasivos adicionales y tratamientos iatrogénicos.

## Data Availability

Se permitirá acceder al material complementario bajo petición a la autora de correspondencia.
